# Functional Changes in the Gut Microbiome Contribute to Transforming Growth Factor β-Deficient Colon Cancer

**DOI:** 10.1128/mSystems.00065-17

**Published:** 2017-09-26

**Authors:** Scott G. Daniel, Corbie L. Ball, David G. Besselsen, Tom Doetschman, Bonnie L. Hurwitz

**Affiliations:** a Department of Molecular and Cellular Biology, University of Arizona, Tucson, Arizona, USA; b The University of Arizona Cancer Center, University of Arizona, Tucson, Arizona, USA; c University Animal Care, University of Arizona, Tucson, Arizona, USA; d Department of Cellular and Molecular Medicine, University of Arizona, Tucson, Arizona, USA; e BIO5 Institute, University of Arizona, Tucson, Arizona, USA; f Department of Agricultural and Biosystems Engineering, University of Arizona, Tucson, Arizona, USA; Mayo Clinic

**Keywords:** *Helicobacter hepaticus*, *Smad3*, bioinformatics, butyrate, colon cancer, gut inflammation, gut microbiome, host-pathogen interactions, metagenomics, metatranscriptomics, polyamines

## Abstract

Colorectal cancer (CRC) is one of the most treatable cancers, with a 5-year survival rate of ~64%, yet over 50,000 deaths occur yearly in the United States. In 15% of cases, deficiency in mismatch repair leads to null mutations in transforming growth factor β (TGF-β) type II receptor, yet genotype alone is not responsible for tumorigenesis. Previous work in mice shows that disruptions in TGF-β signaling combined with *Helicobacter hepaticus* cause tumorigenesis, indicating a synergistic effect between genotype and microbial environment. Here, we examine functional shifts in the gut microbiome in CRC using integrated -omics approaches to untangle the role of host genotype, inflammation, and microbial ecology. We profile the gut microbiome of 40 mice with/without deficiency in TGF-β signaling from a *Smad3* (mothers against decapentaplegic homolog-3) knockout and with/without inoculation with *H. hepaticus*. Clear functional differences in the microbiome tied to specific bacterial species emerge from four pathways related to human colon cancer: lipopolysaccharide (LPS) production, polyamine synthesis, butyrate metabolism, and oxidative phosphorylation (OXPHOS). Specifically, an increase in *Mucispirillum schaedleri* drives LPS production, which is associated with an inflammatory response. We observe a commensurate decrease in butyrate production from *Lachnospiraceae* bacterium A4, which could promote tumor formation. *H. hepaticus* causes an increase in OXPHOS that may increase DNA-damaging free radicals. Finally, multiple bacterial species increase polyamines that are associated with colon cancer, implicating not just diet but also the microbiome in polyamine levels. These insights into cross talk between the microbiome, host genotype, and inflammation could promote the development of diagnostics and therapies for CRC.

**IMPORTANCE** Most research on the gut microbiome in colon cancer focuses on taxonomic changes at the genus level using 16S rRNA gene sequencing. Here, we develop a new methodology to integrate DNA and RNA data sets to examine functional shifts at the species level that are important to tumor development. We uncover several metabolic pathways in the microbiome that, when perturbed by host genetics and *H. hepaticus* inoculation, contribute to colon cancer. The work presented here lays a foundation for improved bioinformatics methodologies to closely examine the cross talk between specific organisms and the host, important for the development of diagnostics and pre/probiotic treatment.

**Author Video**: An author video summary of this article is available.

## Abstract (precis)

Most research on the gut microbiome in colon cancer focuses on taxonomic changes at the genus level using 16S rRNA gene sequencing. Here, we develop a new methodology to integrate DNA and RNA data sets to examine functional shifts at the species level that are important to tumor development. We uncover several metabolic pathways in the microbiome that, when perturbed by host genetics and *H. hepaticus* inoculation, contribute to colon cancer. The work presented here lays a foundation for improved bioinformatics methodologies to closely examine the cross talk between specific organisms and the host, important for the development of diagnostics and pre/probiotic treatment.

## INTRODUCTION

In recent years, colorectal cancer (CRC) ranks as the third most deadly cancer with approximately ~50,000 deaths in the United States alone (1). Chronic intestinal inflammation plays a key role in CRC development, given that patients with inflammatory bowel disease (IBD), ulcerative colitis (UC), or Crohn’s disease (CD) have an increased risk of CRC (2–5). IBD-associated colorectal carcinogenesis is characterized by a sequence of inflammation > dysplasia > carcinoma (reviewed in reference 3). Transforming growth factor β (TGF-β) signaling is one of the key pathways altered in IBD-associated CRC (6–8). TGF-βs are multifunctional cytokines important in diverse biological processes, including development, differentiation, and immune regulation (reviewed in reference 9), yet it is unclear how these processes are involved in colon tumor suppression.

The human TGF-β type II receptor gene (*TGFBR2*) is one of the most frequently mutated genes in IBD-CRCs (10, 11). Previous studies in human CRC cell lines and tumors show that frameshift mutations in the poly(A)_10_ microsatellite region of *TGFBR2* (10–13) result in the loss of TGFβR2 protein production and functional TGF-β signaling (14, 15). Like sporadic and hereditary nonpolyposis colorectal cancer (Lynch syndrome), IBD-CRCs with microsatellite instability have a higher frequency (57 to 76%) of mutations in *TGFBR2*. Consequently, mutations in *TGFBR2* in dysplastic tissues that result in loss of TGF-β signaling play a role in the development of CRC (11).

Mutations in mothers against decapentaplegic (*SMAD*) genes at rates of 3.4% (*SMAD2*), 4.3% (*SMAD3*), and 8.6% (*SMAD4*) in sporadic CRC tumors also disrupt TGF-β signaling (16). SMAD2/3 are receptor activated and bind to SMAD4 (co-SMAD) to form a transcriptional complex. Mutations in *SMAD3* can downregulate *SMAD3* transcription. In inflamed intestinal mucosa samples from patients with active CD and UC, elevated production of SMAD7 (17) inhibits TGFβR1 kinase-mediated phosphorylation of SMAD3 protein and disrupts TGF-β signaling (18). Taken together, mutations in *TGFBR2* or *SMAD2/3*/*4* or elevated SMAD7 levels disrupt TGF-β signaling and accelerate CRC pathogenesis. Understanding how signaling pathways are perturbed in the absence of TGF-β signaling can offer novel strategies to decrease the incidence of CRC in IBD patients with TGF-β signaling deficiency. To investigate TGF-β signaling as it relates to colon cancer, several mouse models have been developed (8, 19–23).

Of the TGF-β-signaling-deficient colon cancer mouse models, the immunocompetent *Smad3* knockout (*Smad3*^−/−^) mouse is the model of choice (23) because the *Tgfb1*^−/−^ *Rag2*^−/−^ model is immunodeficient (20), the TGFβR2-deficient mouse is embryonic lethal (24), and the intestine-specific *Tgfbr2* knockout mouse must be combined with another colon tumor suppressor (8). In both the *Tgfb1*^−/−^ *Rag2*^−/−^ and *Smad3*^*−/−*^ models, colon cancer develops only in conjunction with the presence of gut microbial *Helicobacter* species (21, 22). Interestingly, the potent inflammation-inducing agent dextran sodium sulfate (DSS) in the absence of *Helicobacter* does not induce colon cancer in the *Tgfb1*^−/−^ *Rag2*^−/−^ model (21) and induces only a few late-onset tumors in the *Smad3*^−/−^ model (19). This suggests that inflammation alone in the absence of SMAD3 is not sufficient for tumor development. Consequently, the contribution of *Helicobacter* to tumor development in this model consists of more than just adding inflammatory stress to the colon. Aside from *Helicobacter*, there have been several species identified that are shown to be causative or correlative in the development of colon cancer (Table 1) (25–38). However, no study to date has investigated the functions affected by microbial ecology as a whole.

**TABLE 1:** Bacterial species associated with colon cancer^a^

Name(s)	Relationship	Reference(s)
*Citrobacter rodentium*	Min mice inoculated with this species had a 4-fold increase in colonic tumors. Found only in mice.	25
*Enterococcus faecalis*	Produces superoxide and hydrogen peroxide, both of which can damage DNA in epithelial cells	26, 27
*Clostridium* cluster XVIa (*Clostridium scindens*, *C. hiranonis*, and *C. hylemonae*) and* Clostridium* cluster XI (*C. sordellii*)	Can produce secondary bile acids, such as deoxycholic acid (DOC), that increase tumor burden in wild-type male B6.129PF2/J mice	28, 29
*Acidovorax* species	Associated with increased risk for colon cancer and may act as a pathogen by increased metabolism of nitroaromatic compounds	30
Enterotoxigenic *Bacteroides fragilis*	Produces a toxin that caused colitis and tumors in multiple intestinal neoplasia (Min^Apc716+/−^) mice through an IL-17-dependent pathway	31
*Streptococcus gallolyticus*	Present in approximately 20 to 50% of colon tumors compared to less than 5% of normal tissue in CRC patients. Patients with high counts of *S. gallolyticus* have increased expression of proinflammatory cytokines, including interleukin-1 (IL-1) and cyclooxygenase-2 (COX-2).	32
*Escherichia coli* NC101	Produces genotoxic colibactin that led to increased tumor burden in AOM/*Il10*^−/−^ mice	33
*Fusobacterium nucleatum*	Induces hyperproliferation of human colon cancer cell lines through binding of its FadA adhesin to E-cadherin. Also, promotes immune evasion of tumors through binding of its Fap2 adhesin to TIGIT receptors on immune cells.	34, 35, 37, 38
*Akkermansia muciniphila*	Mucin-degrading species were found in 4-fold-larger amounts in CRC colons than in normal colons	36

a Species names, details of association, and references are listed in chronological order.

Additionally, most metagenomic studies of gut bacteria in colon cancer use 16S rRNA sequencing, which provides only an estimation of taxonomic composition to the genus level. As shown in Table 1, there are certain species that combine with genetic backgrounds to produce colon cancer. Because of this complexity, it is necessary to study gut microbial ecology at the species level and to identify functions causing or preventing CRC.

We hypothesize that a deficiency in TGF-β signaling through loss of SMAD3, combined with the presence of *Helicobacter hepaticus*, alters microbial ecology, leading to functional dysbiosis and colon cancer. To address this, we analyze the mouse gut microbiome in *Smad3*^*+/+*^ and *Smad3*^−/−^ mice in the presence and absence of *H. hepaticus* using a novel approach of integrating metagenomics and metatranscriptomics.

Here, we report several novel findings related to the microbiome. First, we have identified the species, *Lachnospiraceae* bacterium A4, which has decreased RNA counts of butyrate kinase. The family *Lachnospiraceae* has previously been associated with a possible anti-inflammatory role (39–41), but the modality of that role has yet to be elucidated. Our results suggest that this species could be modulating inflammation via butyrate production. The second novelty is the change in RNA counts of polyamine genes arising from several bacterial species. Previous research shows changes in levels of polyamines to be associated with colon cancer (42, 43), but changes to prokaryotic polyamine genes in colon cancer have never been reported. Third, we observe increased RNA counts of lipopolysaccharide (LPS) genes in *Mucispirillum schaedleri*. Although it was previously just associated with inflammation (44–47), our results suggest that *M. schaedleri* is increasing inflammation through increased LPS production. Finally, *H. hepaticus* itself has increased RNA counts of genes involved in oxidative phosphorylation (OXPHOS). This suggests that besides its production of known inflammatory toxins, *H. hepaticus* exerts an oncogenic effect through oxidative damage.

## RESULTS

### Study overview.

**FIG 1:**
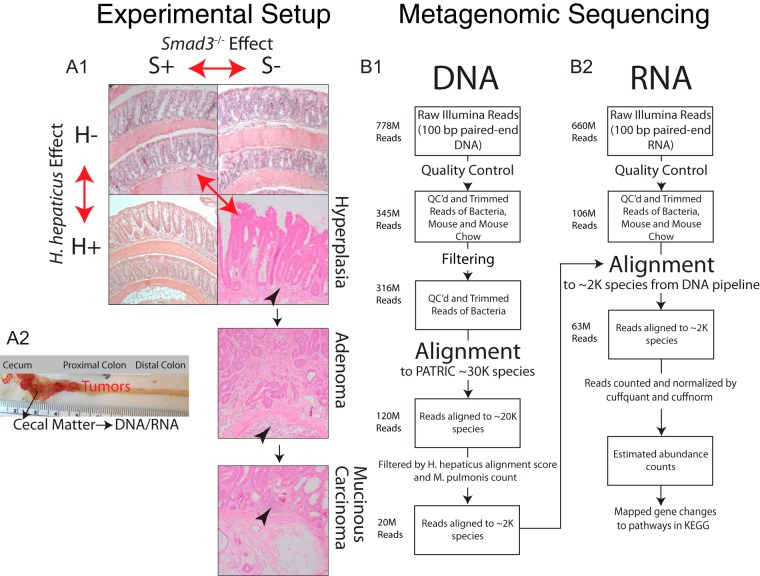
Materials and methods flow chart. (A1) Four groups of mice were used for DNA/RNA extraction: a mouse that was *Smad3*^*+/+*^ and *H. hepaticus* negative (S+H−) (top left), a mouse that was *Smad3*^*−/−*^ and *H. hepaticus* negative (S−H−) (top right), a mouse that was *Smad3*^*+/+*^ and *H. hepaticus* positive (S+H+) (bottom left), and a mouse that was *Smad3*^*−/−*^ and *H. hepaticus* positive (S−H+) (bottom right, three panels from one histological section). These three panels display the types of lesions found in the S−H+ mice: hyperplasia, adenoma, and mucinous carcinoma. Inflammatory infiltrates are indicated by arrowheads. Four out of 10 mice in the S−H+ group had tumors at the time of sacrifice. The three double-headed red arrows indicate the *Smad3*^*−/−*^, *H. hepaticus*, and combined effects. (A2) Dissection of mouse large intestine from the S−H+ group showing tumor locations in dotted red lines and site of cecal matter removal. (B1) Flow chart showing number of reads at each step of DNA analysis. Reads were aligned to PATRIC (48) with Taxoner64 (95). The last step of the DNA analysis feeds into the second step of the RNA analysis. (B2) Flow chart showing number of reads at each step of RNA analysis. Reads were aligned with Bowtie2 (97) and quantified/normalized with cuffquant/cuffnorm, part of the Cufflinks suite of tools (99).

Cecal samples from 40 mice were pooled into four comparison groups: (i) *Smad3*^+/+^*/H. hepaticus* negative (wild type; S+H−), (ii) *Smad3*^*−*/−^/*H*. *hepaticus* negative (*Smad3*^*−/−*^ only; S−H−), (iii) *Smad3*^+/+^/*H. hepaticus* positive (*H. hepaticus* only; S+H+), and (iv) *Smad3*^*−*/−^/*H*. *hepaticus* positive (combined; S−H+). Figure 1A summarizes each comparison group where cecal samples from 10 mice were pooled per group (1:1 male/female ratio). Wild-type (S+H−), *H. hepaticus*-only (S+H+), and *Smad3*^*−/−*^-only (S−H−) mice show little histologically evident inflammation and no cancer or precancerous hyperplastic lesions. However, mice with the combined *Smad3*^*−/−*^ and *H. hepaticus* inoculation (S−H+) show significant inflammation, and 40% of mice (2 males and 2 females) develop tumors in the cecum and proximal colon by 6 months of age. One animal had a large cecal tumor and was 9 months of age. The literature shows that, in animals with various combinations of *Helicobacter* species, *Smad3*^*−/−*^ mice developed tumors with 22 to 66% penetrance over a 30-week period (22). Figure 1B shows a flow chart of bioinformatics methods. After quality control (QC) and filtering for host, 316 million reads remain, of which 120 million align to known bacterial genomes in the PATRIC database (48). After filtering based on positive and negative controls, 20 million reads align to 1,944 bacteria with high confidence, representing our “gold standard” genomes (details in Materials and Methods). Overall, ~60% of RNA reads (63 million of 106 million RNA reads) map to these gold standard genomes. Thus, although the DNA mapping to the gold standard genomes represents only ~7% of the total data set (20 million DNA reads of 316 million map to gold standard genomes), these species are functionally dominant based on RNA read recruitment.

### Functional shifts in the microbiome.

**FIG 2:**
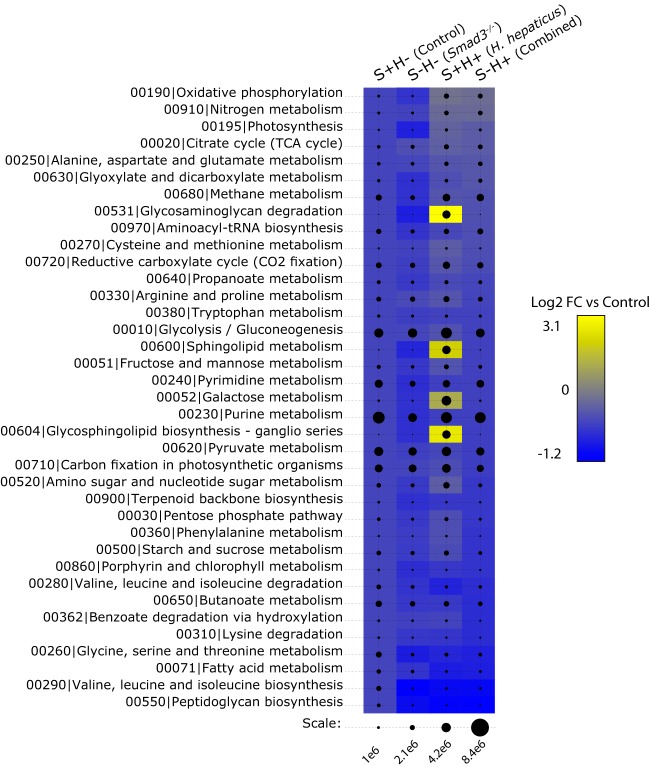
Sum of estimated expression by KEGG pathway. Bubble chart of RNA count differences between sample groups among various KEGG pathways (102). The scale at bottom shows estimated RNA counts as outputted by cuffquant and cuffnorm (part of the Bowtie2/Cufflinks suite of tools). Labels at top show the sample groups. The heat map behind the bubbles shows log_2_ fold changes (log_2_ FC) of pathways for each of the effects. Fold changes are mapped to a blue-yellow color spectrum with bright yellow having the greatest increase in RNA counts and bright blue having the greatest decrease in RNA counts under the given condition versus control. Color behind the control bubbles represents no change and is provided for reference.

To guide our functional analysis, we examine the top 37 pathways (Fig. 2) ranked by overall estimated RNA counts, where each pathway contains total RNA counts above the pathway mean count (all 140 pathway counts are available at https://doi.org/10.6084/m9.figshare.5328700). Then, we examine pathways that either have previous links to CRC or have the greatest changes in RNA counts among groups. Previous literature shows that butyrate, polyamine, and OXPHOS pathways are important in colorectal cancer and/or inflammation (49–51), and they rank 8th, 9th, and 18th, respectively, in these 37 pathways (LPS is 73rd but is included because of its known role in inflammation [reviewed in reference 52]). In each case, we examine pathways gene by gene to highlight the greatest changes in RNA counts and potential points of enzymatic flux changes. Additionally, we focus on significant changes to RNA counts in genes by sample type using the following comparisons: S−H− versus S+H−, S+H+ versus S+H−, and S−H+ versus S+H− (here referred to as *Smad3*^*−/−*^ effect, *H. hepaticus* effect, and combined effect, respectively). The combined effect represents the contributions of both the loss of SMAD3 and *H. hepaticus* inoculation. Results are split into two sections: first, pathways that are changed by the *Smad3*^*−/−*^ and combined but not *H. hepaticus* effects, and second, pathways that are changed by the *H. hepaticus* and combined but not *Smad3*^*−/−*^ effects.

### *Smad3*^*−/−*^ and combined effects on bacterial pathways. (i)* Lachnospiraceae* bacterium A4 is responsible for decreased RNA counts of butyrate kinase.

In the normal colon, butyrate is a primary energy source for colon mucosal epithelial cell growth through oxidative rather than glucose metabolism (53, 54). In colon tumors, however, aerobic glycolysis of glucose is the primary source of energy, causing butyrate to accumulate in the nucleus, where it becomes a histone deacetylase (HDAC) inhibitor (55–58) under Warburg conditions (59). HDAC inhibition promotes cell cycle arrest and apoptosis through p21 expression (60) and inhibits NF-κB activation by decreasing the proteasome activity responsible for IκB degradation (61). In addition, butyrate also increases T-cell regulation (62). Butyrate, therefore, has both antitumor and anti-inflammatory activities, making it an effective anti-UC therapy (63). It seems reasonable, then, that in the context of colon cancer decreased colonic levels of butyrate would promote cancer cell growth and stimulate inflammation.

**FIG 3:**
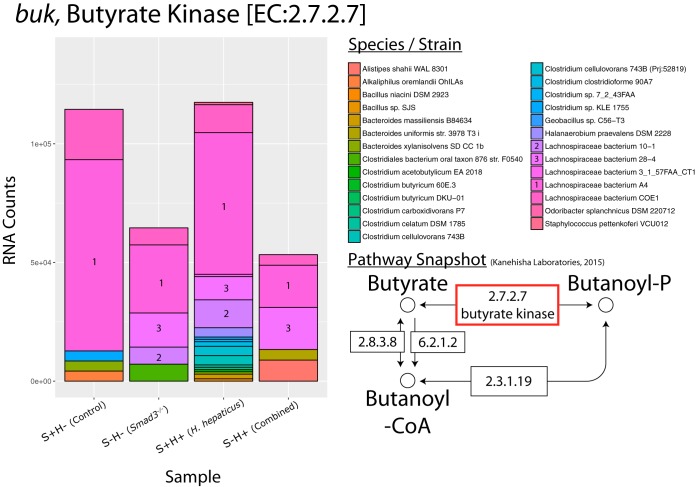
Species contribution to genes in butanoate (butyrate) metabolism. RNA count changes for *buk* (butyrate kinase) (EC 2.7.2.7), categorized by species/strain contribution. The *y* axis shows RNA counts, and the *x* axis shows sample groups. Species with majority contributions to count bars are numbered for clarity. Bottom right shows partial pathway with a red box to highlight gene. Partial pathway adapted from KEGG pathway database (102).

**FIG 4:**
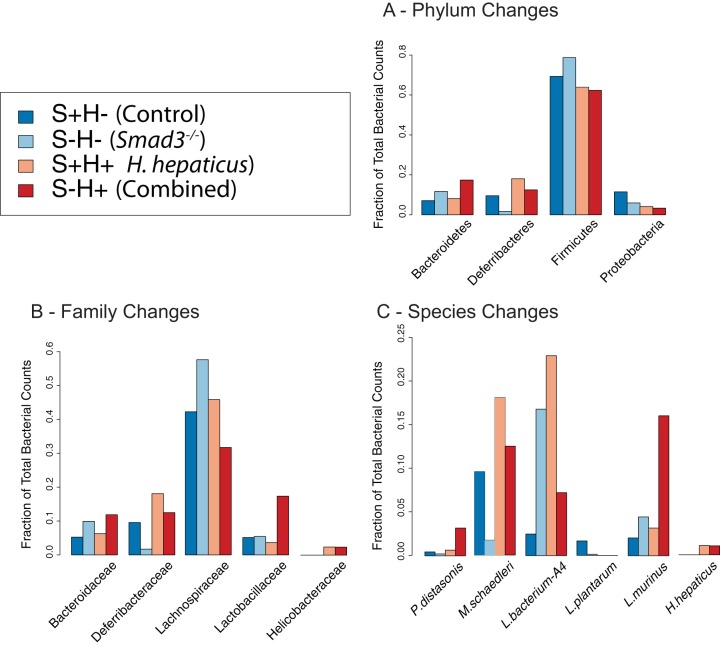
Taxonomic changes at phylum, family, and species levels. Changes in fractions of total bacterial counts at different taxonomic levels for the S+H− (Control), S−H− (*Smad3*^*−/−*^), S+H+ (*H. hepaticus* only), and S−H+ (Combined) groups. (A) Changes for the phyla of *Bacteroidetes*, *Deferribacteres*, *Firmicutes*, and *Proteobacteria*. (B) Changes for the families of *Bacteroidaceae*, *Deferribacteraceae*, *Lachnospiraceae*, *Lactobacillaceae*, and *Helicobacteraceae*. (C) Changes for the species of *Mucispirillum schaedleri*, *Lachnospiraceae* bacterium A4, *Lactobacillus plantarum*, *Lactobacillus murinus*, *Parabacteroides distasonis*, and *Helicobacter hepaticus*.

In the butyrate metabolism pathway, we see a decrease in RNA counts for butyrate kinase (*buk*) with a log_2_ fold chance (FC) of −1.1 in the combined effect (Fig. 3; also see https://doi.org/10.6084/m9.figshare.5047477 and https://doi.org/10.6084/m9.figshare.5325136). This enzyme is important because it is the last step in the production of butyrate. This decrease in *buk* may be a genotypic effect, given that the *Smad3*^*−/−*^ effect shows a similar decrease. In contrast, the *H. hepaticus* effect shows a slight increase. Interestingly, the main bacterial species whose decrease in abundance contributes to changes in butyrate kinase is *Lachnospiraceae* bacterium A4, a relatively understudied species in the realm of bacterial butyrate producers. Other contributors include members of the *Lachnospiraceae* family, *Lachnospiraceae* bacterium 10-1 and *Lachnospiraceae* bacterium 28-4. Along with decreased RNA counts of *buk*, we see a decrease in the abundance of the *Lachnospiraceae* family and the *Firmicutes* phylum (Fig. 4; see also https://doi.org/10.6084/m9.figshare.5051722) but a surprising increase in the population of *Lachnospiraceae* bacterium A4 (2.92-fold) in the combined effect. However, despite the increase in population of *Lachnospiraceae* bacterium A4, the RNA counts show a decrease in *buk* expression by this bacterium, suggesting a downregulation of *buk*. Changes to the abundance of bacteria in the *Lachnospiraceae* family and *Firmicutes* phylum represent an avenue where host genotype may contribute more to microbial ecology than inoculation with *H. hepaticus*.

### (ii) Multiple species have increased RNA counts for genes producing putrescine and spermidine.

Putrescine is known to be required for early development, as a lack of it causes cell apoptosis and prenatal death in mice (64). Spermidine was shown to be required for posttranslational modification of eukaryotic initiation factor 5A (eIF5A), which is required for growth in a range of species (65, 66). Large amounts of polyamines, thought to be derived from diets high in red meat (67), are associated with severity of colorectal cancer (43). Also, when patients are in remission, their polyamine levels decrease (43). While there have been numerous studies on the link between polyamines and colorectal cancer in eukaryotic cells, no study to date has shown a prokaryotic contribution.

**FIG 5:**
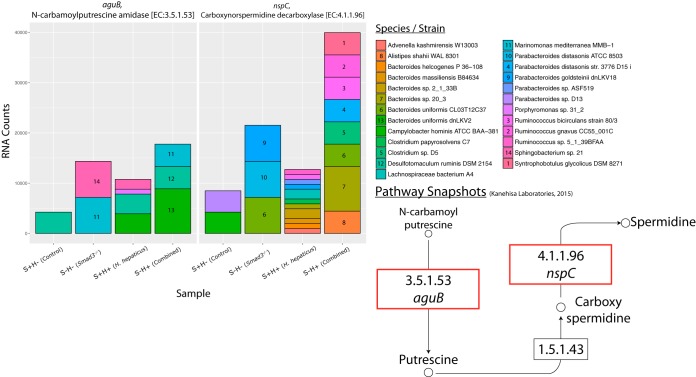
Species contribution to genes in arginine and proline metabolism. RNA count changes for *aguB* (*N*-carbamoylputrescine amidase) (EC 3.5.1.53) and *nspC* (carboxynorspermidine decarboxylase) (EC 4.1.1.96), categorized by species/strain contribution. The *y* axis shows RNA counts, and the *x* axis shows sample groups. Species with majority contributions to count bars are numbered for clarity. Bottom right shows partial pathway with a red box to highlight genes. Partial pathway adapted from KEGG pathway database (102).

Two genes that have higher RNA counts in the arginine and proline pathway are *N*-carbamoylputrescine amidase (*aguB*) and carboxynorspermidine decarboxylase (*nspC*), by a log_2_ FC of 2.07 and 2.24, respectively, in the combined effect (Fig. 5; see also https://doi.org/10.6084/m9.figshare.5051728 and https://doi.org/10.6084/m9.figshare.5325151). *aguB* and *nspC* are genes for enzymes responsible for one of the reactions that produce the polyamines putrescine and spermidine, respectively. Similarly to *buk*, these genes have different RNA counts in the *Smad3*^*−/−*^ and combined effects but little difference in the *H. hepaticus* effect.

For *aguB*, in the combined group (S−H+), the species *Marinomonas mediterranea* MMB-1, *Desulfotomaculum ruminis* DSM 2154, and *Bacteroides uniformis* dnLKV2 are responsible for the majority of the expression. For *nspC*, in the combined group, a majority of the RNA counts are represented by a diverse group of species from the genera *Bacteroides*, *Clostridium*, *Ruminococcus*, and *Alistipes*. *Parabacteroides distasonis* has a small contribution to the RNA counts but has a dramatic change in abundance. An interesting point is that there is no single dominant species that is responsible for the upregulation of these genes.

In terms of taxonomic shifts, *P. distasonis* has nearly a 10-fold increase in the combined effect. *P. distasonis* has been previously associated with inflammation in a DSS mouse model of colitis (68). The family *Bacteroidaceae*, of which *Bacteroides uniformis* is a member, is increased by 1.89-fold in the *Smad3*^*−/−*^ effect and 1.20-fold in the *H. hepaticus* effect (Fig. 4; see also https://doi.org/10.6084/m9.figshare.5051722). In the combined effect, we see a synergistic effect in *Bacteroidaceae* with a 2.26-fold increase. We see similar fold changes at the phylum level of *Bacteroidetes*.

### *H. hepaticus* and combined effects on bacterial pathways. (i)* M. schaedleri*, a core member of the mouse gut bacteria, is a major contributor to increased RNA counts for LPS genes in *H. hepaticus*-only mice.

Gram-negative bacteria use LPSs as structural molecules to make up their outer membrane, and they are released whenever the bacteria divide or die. In addition to lipopeptides and flagellins, LPSs are known to be signaling molecules for inflammatory pathways. In particular, LPSs have been shown to activate the cluster of differentiation 14/myeloid differentiation 2/Toll-like receptor 4 (CD14/MD2/TLR4) receptor pathway (69). This ultimately leads to increased transcription of proinflammatory cytokines such as tumor necrosis factor alpha (TNF-α) and interleukin-6 (IL-6). As already discussed, there are strong correlations between the presence of inflammatory conditions and the progression of colorectal cancer; thus, we examined LPS gene signatures in our data set.

**FIG 6:**
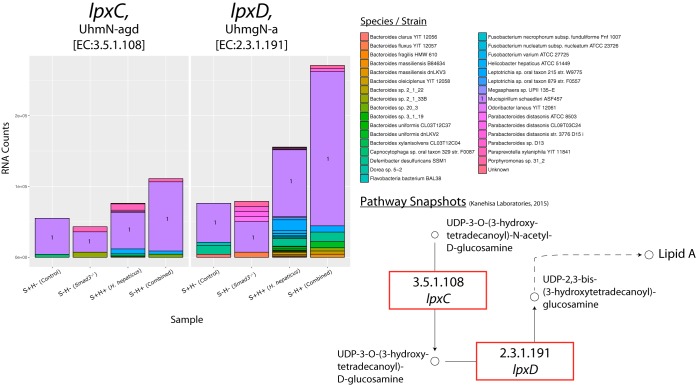
Species contribution to *lpxC* and *lpxD* in LPS biosynthesis. RNA count changes for *lpxC* [UDP-3-*O*-(3-hydroxymyristoyl) *N*-acetylglucosamine deacetylase] (EC 3.5.1.108) and *lpxD* [UDP-3-*O*-(3-hydroxymyristoyl) glucosamine *N*-acyltransferase] (EC 2.3.1.191), categorized by species/strain contribution. The *y* axis shows RNA counts, and the *x* axis shows sample groups. Species with majority contributions to count bars are numbered for clarity. Bottom right shows partial pathway with a red box to highlight genes. Partial pathway adapted from KEGG pathway database (102).

Several LPS genes have increased RNA counts in the *H. hepaticus* and combined effects: 3-deoxy-d-manno-octulosonic-acid transferase (*kdtA*), UDP-3-*O*-(3-hydroxymyristoyl) *N*-acetylglucosamine deacetylase (*lpxD*), and UDP-3-*O*-(3-hydroxymyristoyl) glucosamine *N*-acyltransferase (*lpxC*). In this same order, these genes have log_2_ FCs of 2.1, 1.8, and 1 in the combined effect (Fig. 6; see also https://doi.org/10.6084/m9.figshare.5051734 and https://doi.org/10.6084/m9.figshare.5325154). There is little change or a decrease in the *Smad3*^*−/−*^ effect for these genes.

In terms of species contribution, a surprising finding is that *M. schaedleri* ASF457 is responsible for a majority of the RNA counts of *lpxC and lpxD*. This is interesting because *M. schaedleri* is associated with inflammatory pathways (44–47), but studies have not shown which genes may be involved. Mirroring the RNA count changes, *M. schaedleri* abundance is increased in the *H. hepaticus*-only and combined effects by 1.89-fold and 1.31-fold, respectively, while there is a decrease in the *Smad3*^*−/−*^ effect (Fig. 4; see also https://doi.org/10.6084/m9.figshare.5051722).

### (ii) Increased RNA counts of LPS genes in bacteria correlate significantly with host TLR gene expression.

**FIG 7:**
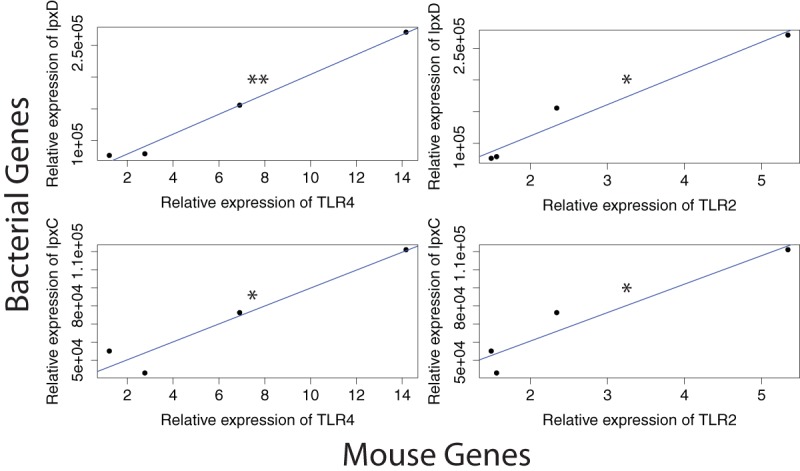
Correlation of mouse gene expression with bacterial gene expression. RNA expression correlation between mouse TLR genes and bacterial genes in the LPS pathway. Expression in arbitrary units due to normalization. Mouse gene expression based on qRT-PCR data; bacterial gene expression based on transcriptome sequencing from this study. *P* values: *, *P* < 0.05; **, *P* < 0.01; ***, *P* < 0.001.

As mentioned above, LPSs activate inflammation via the CD14/MD2/TLR4 receptor pathway. To determine whether TLR receptor activity of *Smad3*^*−*/−^/*H*. *hepaticus*-positive mice could correlate with increases in bacterial LPS production, we measured the mucosal epithelial expression of these genes using real-time quantitative reverse transcription PCR (qRT-PCR). We find that *Tlr2* and *Tlr4* expression both significantly correlate with the increased counts of bacterial *lpxC* and *lpxD* (Fig. 7).

### (iii) *H. hepaticus* has increased RNA counts of key genes in the OXPHOS pathway.

It has been established that cancer cells prefer aerobic glycolysis, but it has also been shown that they still contain active mitochondria to produce a portion of their ATP (70, 71). However, although oxidative phosphorylation (OXPHOS) may be taking place in the epithelial cells, less is known about the microbiome’s metabolic activities. It seems plausible that, as in cancer cells, a preference for aerobic glycolysis or OXPHOS in microbial cells will have an effect on the tumor microenvironment. OXPHOS, aside from being indicative of proliferating bacterial cells, increases the amount of reactive oxygen and nitrogen species (RONS), which are known to damage DNA, RNA, and proteins (72–74).

**FIG 8:**
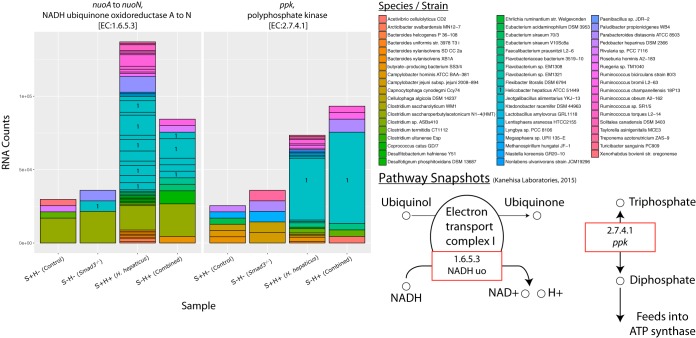
Species contribution to genes in oxidative phosphorylation. RNA count changes for *nuoA* to *nuoN* (NADH ubiquinone oxidoreductase A to N) (EC 1.6.5.3) and *ppk* (polyphosphate kinase) (EC 2.7.4.1), categorized by species/strain contribution. The *y* axis shows RNA counts, and the *x* axis shows sample groups. Species with majority contributions to count bars are numbered for clarity. Dividing lines within species represent multiple subunits for that gene. Bottom right shows partial pathway with a red box to highlight genes. Partial pathway adapted from KEGG pathway database (102).

In our study, we see a log_2_ FC of 1 and 1.5 for *nuo* (NADH ubiquinone oxidoreductase [NADHuo]) in the combined and *H. hepaticus* effects, respectively (Fig. 8; see also https://doi.org/10.6084/m9.figshare.5051737 and https://doi.org/10.6084/m9.figshare.5325157). NADHuo is part of the first electron transport complex for the production of ATP. Likewise, in these same effects, we see a log_2_ FC of 1.3 and 1 for *ppk* (polyphosphate kinase), respectively. Polyphosphate kinase prepares inorganic triphosphate for feeding into ATP synthase. It is true that there are other genes in the pathway that are lowered in RNA counts, but the pathway overall has increased counts in the combined effect (Fig. 2 and https://doi.org/10.6084/m9.figshare.5328700).

The species primarily responsible for the increase in oxidative phosphorylation is *H. hepaticus*. Not surprisingly, given that the S+H+ and S−H+ mice were inoculated with the species, we see the contribution of *H. hepaticus* to OXPHOS genes in those groups. There is a small contribution in the S−H− group for *nuo*, but this could be due to sequencing/alignment error of a closely related species.

## DISCUSSION

Colon cancer is a multifactorial disease affected by host genetics, resident gut bacterial species, diet, and inflammation. In terms of host genetics, 10 to 15% of colon cancer patients have mutations in TGF-β signaling genes, but their function as tumor suppressors and interaction with gut bacteria is unclear. In mouse colon cancer models in which growth has been measured, no loss of growth control was attributable to the loss of TGF-β signaling under pretumor conditions (8, 20). However, a role for TGF-β signaling in both the differentiation and inflammatory states of colon tumors was revealed in a comparative microarray study of several mouse colon tumor models (75). Since TGF-β signaling is known to be an important regulator in immune tolerance and T-cell homeostasis (reviewed in reference 76), it is likely that its absence could function, at least in part, to exacerbate an inflammatory response in the mouse colon.

The propensity of colorectal cancer having an inflammatory component suggests that microbial dysbiosis may result from tumor-suppressing activities of TGF-β. Here, we report changes to microbial functions in a TGF-β signaling-deficient colon cancer model.

First, a key butyrate gene, *buk*, is found to have reduced RNA counts in the *Smad3*^*−/−*^ and combined effects. This agrees with multiple studies that show butyrate to be crucial for proper maintenance of the colonic epithelium (54). It is well known that butyrate is the primary energy source of colonocytes; moreover, several studies have shown that in high concentrations (~5 mM), butyrate is a potent HDAC inhibitor, resulting in expression of several genes involved in cancer or inflammation: cyclin-dependent kinase inhibitor 1A (p21WAF1/Cip1), mucin 2 (MUC-2), testin LIM domain protein (TES), and hypoxia-inducible factor 1 (HIF-1) (55, 56, 58, 59). Additionally, it has been shown that it can stimulate histone acetyltransferase (HAT) activity at lower concentrations (~0.5 mM) (59). This happens when butyrate is converted to citrate in the tricarboxylic acid (TCA) cycle which combines with ATP citrate lyase (ACL) to produce acetyl coenzyme A (acetyl-CoA). This important coenzyme then acts as an acetyl group donor for various HATs. While we cannot conclude that the reduction in RNA counts of butyrate genes is a cause or effect of the proinflammatory, cancerous environment, the correlation is consistent with the literature.

In our study, *Lachnospiraceae* bacterium A4, a member of the *Firmicutes* phylum, decreases in abundance in the combined and *Smad3*^*−/−*^ effects. This species, which has been little studied, belongs to the *Lachnospiraceae* family of bacteria, of which some literature suggests that it may play an anti-inflammatory role. For example, one study shows that inoculation with an isolate of *Lachnospiraceae* decreases disease severity of chronic *Clostridium difficile* infection in mice (39). Additionally, 16S rRNA studies of human fecal samples from IBD patients have revealed smaller amounts of *Lachnospiraceae* at the genus level (40, 41). One could easily hypothesize that butyrate supplements or probiotic *Lachnospiraceae* would slow cancer growth and reduce inflammation in our cancer model.

Second, we observe an upshift in RNA counts for genes involved in the production of putrescine and spermidine. Like the butyrate gene changes, this occurs in the SMAD3 and combined effects. Changes to polyamine genes are interesting because it has been known since the 1960s that polyamines are increased in rapidly proliferating tissues (77). More recently, it has been discovered that polyamines can also affect protein translation. Specifically, spermidine can be modified to the unique amino acid hypusine, which is the only known amino acid to modify the eukaryotic initiation factor EIF5A (66). This translation factor is not strictly required for translation in general, but it seems to prefer transcripts with polyproline motifs (78, 79). Additionally, it has been shown that blocking the production of hypusine or the modification of EIF5A by hypusine leads to reduced gene translation of growth promoters RhoA/ROCK1 (80). Also, increased polyamine levels in colon cancer patients correlate with severity of disease (42, 43). The novel finding here is that gut bacteria are at least possibly responsible for increased polyamines. It is plausible that the polyamines are being actively exported to the epithelial cells, as such transporters exist for both prokaryotes and eukaryotes (81–83). Further studies will need to be done to measure changes to polyamine levels as well as RNA counts of eukaryotic polyamine genes. Consequently, not just diet but also dysbiosis in the gut microbiome may result in increased polyamine uptake by colon mucosal epithelial cells.

Although not in the list of top pathways, our third focus was LPS biosynthesis because it has been known to produce an inflammatory response for more than a century (although LPSs were termed “endotoxins” before discovery of their structure) (84). Indeed, we find an increase in bacterial genes in the LPS pathway for both the *H. hepaticus*-only and combined effects. Intriguingly, it is not *H. hepaticus* itself that is responsible for the increased RNA counts, it is *M. schaedleri*. Though *M. schaedleri* has been associated with inflammation, its contribution is not known. It should be noted that this species is in the set of core bacteria given to mice in gnotobiotic models (85, 86). Our data show that inoculation with *H. hepaticus* correlates with an increased abundance of *M. schaedleri* that may result in a shift to a proinflammatory state.

In our fourth focus, we find a colon epithelial cell response consistent with increased bacterial LPS production. By qRT-PCR on colon mucosal epithelial mRNA, *Tlr4* and *Tlr2* are shown to be upregulated in all effects compared to control, and this significantly correlates with increased RNA counts of bacterial *lpxC* and *lpxD*. Accordingly, LPS has been shown to activate the proinflammatory NF-κB pathway through the CD14/MD2/TLR4 complex (69, 87). On the other hand, *Helicobacter* spp. have been shown to activate the TLR2 receptor, possibly explaining its upregulation in the mice (88).

Our fifth focus, OXPHOS, shows the most change in RNA counts. This is surprising given the normally anaerobic environment of the colon; increased rates of OXPHOS would imply an aerobic environment. Nevertheless, there are increased counts of *nuo* and *ppk* for the *H. hepaticus* and combined effects. Importantly, the species mainly responsible for this increase is *H. hepaticus*, which may be rapidly growing and depleting the environment of available oxygen. More importantly, an increase in OXPHOS points to an increase in RONS. It could be that the RONS produced by *H. hepaticus* and other species are causing oxidative damage to DNA, RNA, or proteins that leads to a cancerous state. Even though both SMAD3 deficiency and *H. hepaticus* inoculation are required for colon cancer in this model, the OXPHOS pathway is changed more by *H. hepaticus* than by SMAD3 deficiency.

It is important to note that *Lactobacillus plantarum* does not appear to be involved in altering butyrate, polyamine, LPS, or OXPHOS levels, yet it is undetectable in the mice with *H. hepaticus* (Fig. 4; see also https://doi.org/10.6084/m9.figshare.5051722). The dysbiotic environment produced by *H. hepaticus* is incompatible with *L. plantarum* through unknown mechanisms that may involve nutrient competition, susceptibility to toxins, or other environmental factors. Other studies have shown that adding *L. plantarum* reduces tumor size and burden in rats and inhibits survival of cancer stem cells (89, 90). On the other hand, *Lactobacillus murinus* increases in all effects compared to control. This is the first report that links *L. murinus* to inflammation or colon cancer.

In summary, loss of SMAD3 is associated with changes in bacterial RNA counts in the butyrate and polyamine synthesis pathways. And, with the addition of *H. hepaticus*, we see an increase in LPS and OXPHOS pathways’ RNA counts, suggesting an increase in the proinflammatory and free radical status of the colonic epithelium but without histological evidence of inflammation (Fig. 1A). Either of these changes alone is not sufficient to promote carcinogenesis. Rather, it takes their combination and possibly a reduction of probiotic species to reach the “tipping point” for tumorigenesis. The results of this study emphasize the multifactorial nature of colon cancer and how the microbiome may have a profound impact on the cancer microenvironment. This lends credence to the idea that changes in microbial ecology as well as in host genotype must be taken into consideration when examining the causes of colon cancer.

## MATERIALS AND METHODS

### Animal husbandry.

*Smad3*^−/−^ mice (129/Sv) generated previously (23) were obtained from Jackson Laboratories and maintained in a specific-pathogen-free (SPF) facility under a University of Arizona IACUC protocol. Sentinel mice were routinely screened for pathogens. Homozygous *Smad3*^−/−^ and *Smad3*^+/+^ mice were generated by breeding heterozygous animals.

### PCR genotyping.

The genotype of newborn pups from double heterozygous mating was determined by PCR amplification of tail DNA and size fractionation on agarose gels (20).

### *Helicobacter* culture, infection, and detection.

A pure culture of *H. hepaticus* was received from Craig Franklin (University of Missouri) and was suspended in brucella broth on tryptic soy agar supplemented with 5% sheep blood (Hardy Diagnostics) and incubated in a microaerophilic chamber at 37°C for 48 h. Later, the culture was resuspended in brucella broth and allowed to grow for another 48 h. Five breeding pairs of 1- to 3-month-old heterozygous *Smad3*^*+/−*^ mice were inoculated with ~10^8^ *H. hepaticus* organisms by direct introduction using a 1.5-in. feeding needle. Control animals of five breeding pairs, 1- to 3-month-old heterozygous *Smad3*^*+/−*^ mice, were inoculated with equal amounts of brucella broth. A total of 3 inoculations for each mouse was completed at 24-h intervals. Animals were then checked for *H. hepaticus* infection by PCR analysis of fecal matter with *H. hepaticus*-specific primers as described earlier (91). Infected animals then were bred together. All animals in subsequent generations developed chronic infection by spontaneous parental/fecal contact without additional inoculation. To minimize cross contamination, uninfected and infected animals were housed in different buildings.

### Tissue collection and staining.

Mice were euthanized by IACUC-approved cervical dislocation. The cecum and colon were dissected free from the mesenchyme. All tissue sections shown in the figure(s) were from the cecum, and staining was done with hematoxylin and eosin. The cecum and colon were opened longitudinally, and contents were collected according to location. Cecal content, proximal colon content, and distal colon content were placed in individual tubes and flash frozen in liquid nitrogen. All samples were stored at −80°C. Only cecal content was sent for sequencing.

### DNA/RNA sequencing and quality control (including filtering).

Sequencing was done at the University of Arizona Genomics Core (UAGC). DNA was extracted using an in-house lysozyme extraction protocol. The libraries were built with Illumina TruSeq DNA kits (Illumina, San Diego, CA). RNA was ribodepleted using both eukaryotic and prokaryotic RiboMinus kits (Thermo Fisher, Waltham, MA). The RNA libraries were built with the Illumina TruSeq RNA kits. Ten mice from each group were pooled and run on two lanes using an Illumina HiSeq 2000/2500 machine. DNA/RNA reads were 2 × 100-bp paired-end reads, and the average insert size was ~325 bp for DNA and ~225 bp for RNA. After sequencing, adapter sequences were trimmed from raw data before being downloaded to the University of Arizona High-Performance Computing (UA HPC) center. Quality control (QC) was done using a custom pipeline using the programs SolexaQA++ (92) and fastx_clipper from the FASTX suite of tools (93) (https://github.com/hurwitzlab/fizkin). After QC, DNA reads were filtered for mouse and mouse chow (including yeast, barley, soy, wheat, and corn) using jellyfish (94), a kmer frequency counting tool. Filtering was done based on the assumption that reads coming from mouse or mouse chow will have similar kmer frequencies as the source genomes. Therefore, reads that had kmer modes of 2 or greater in comparison to the mouse or mouse chow were considered “rejected” and filtered from downstream analysis. Since quality control and filtering may have eliminated mate pairs, reads were reconstituted into new fastq files: two files for forward and reverse paired-end reads and two files for single-ended reads (those that lost their mates, either forward or reverse).

### DNA alignment.

Alignment was done with Taxoner64 version 0.1.3 (95) against the ~30,000 bacterial and archaeal genomes in PATRIC (48) (genomes downloaded on 5 September 2015 from https://www.patricbrc.org/) using the parameters “-A --very-sensitive-local” (https://github.com/hurwitzlab/taxoner-patric). Results were then filtered with a minimum alignment score of 131 (the average alignment score for *H. hepaticus*, our positive control) and a minimum count of ~138 (the average count of *Mycoplasma pulmonis*, a pathogen that is a negative control since the facility is specific pathogen free [SPF]). A hierarchical pie chart of species composition (https://doi.org/10.6084/m9.figshare.5051722) was constructed using KronaTools (96).

### RNA alignment.

Alignment was done with Bowtie2 version 2.2.6 (97) for aligning against bacterial genomes and TopHat version 2.1.1 (98) for aligning against the mouse genome (Mus_musculus GRCm38 dna_rm primary_assembly fa from Ensembl) (https://github.com/hurwitzlab/bacteria-bowtie). RNA coverage of mouse genome was ~3-fold on average (data not shown). Given this, we excluded mouse results from further analysis. Bacterial genomes were composed of the ~2,000 genomes that passed filtering criteria in the “DNA alignment” step. Additional filtering of RNA for mouse and mouse chow was not necessary due to the use of the selected bacterial genomes.

### Differential gene expression.

To calculate bacterial gene expression, cuffquant was used with the parameters “-M rRNAGFF—no-length-correction,” and cuffnorm (99) was used with default parameters. No length correction was used in our study because we were interested in comparing genes across samples and not within samples (where gene length correction would be necessary [100]). Postprocessing of abundance counts and plotting/heat map generation was done with R version 3.2.2 (101) and Excel version 14.4.0 for Mac (Microsoft Corp., Redmond, WA).

### Pathway mapping and species contribution.

To assign gene products to pathways, annotation information was downloaded from PATRIC (RefSeq.cds.tab files). Once each gene was annotated with pathways, sums were calculated for each gene and each pathway among species and samples. See the figure legends for more details. The bubble chart (Fig. 2) was created using custom R and perl scripts.

### Isolation of RNA from tissue and cDNA synthesis (for qRT-PCR).

Total RNA from individual frozen tissue samples was isolated using TRI reagent (Molecular Research Center, Cincinnati, OH). RNA was treated with RNase-free DNase I (Qiagen, Valencia, CA) and purified using a Qiagen RNeasy minikit. RNA was reverse transcribed using an iScript cDNA synthesis kit (Bio-Rad, Hercules, CA).

### Primer design and SYBR green qRT-PCR.

qRT-PCRs were performed using a Light Cycler 480 (Roche, Basel, Switzerland) with 50 to 100 ng of cDNA template. At least one primer per pair was designed across exon-intron boundaries to prevent coamplification of genomic DNA; the sizes of the products range from 50 to 150 bp. For each gene, threshold cycle (*C*_*T*_) values were normalized to corresponding β-actin or glyceraldehyde-3-phosphate dehydrogenase (GAPDH), and relative expression was determined by the 2−ΔΔ*CT* method.

### Correlation of bacterial RNA count with mouse RNA count.

Since the bacterial RNA count had only a single data point for each group, median expression values were used from the mouse qRT-PCR data. Using these values, Pearson’s product-moment correlation tests were run using default parameters. The lm() command in R was used to construct linear models and plot regression lines in Fig. 7.

### Data availability.

All raw DNA/RNA reads are available under BioProject number PRJNA379709. 
